# Tsallis Entropy for Assessing Spatial Uncertainty Associated with Mean Annual Runoff of Quaternary Catchments of the Middle Vaal Basin in South Africa

**DOI:** 10.3390/e22091050

**Published:** 2020-09-19

**Authors:** Masengo Ilunga

**Affiliations:** Department of Civil Engineering and Chemical Engineering, College of Science, Engineering and Technology, Private Bag X6, Florida Campus, University of South Africa, Johannesburg 1710, Florida, South Africa; ilungm@unisa.ac.za; Tel.: +27-114-712-791

**Keywords:** entropy, q-uncertainty/information deviation, runoff, catchment, iso q-information deviation maps

## Abstract

This study assesses mainly the uncertainty of the mean annual runoff (MAR) for quaternary catchments (QCs) considered as metastable nonextensive systems (from Tsalllis entropy) in the Middle Vaal catchment. The study is applied to the surface water resources (WR) of the South Africa 1990 (WR90), 2005 (WR2005) and 2012 (WR2012) data sets. The q-information index (from the Tsalllis entropy) is used here as a deviation indicator for the spatial evolution of uncertainty for the different QCs, using the Shannon entropy as a baseline. It enables the determination of a (virtual) convergence point, zone of positive and negative uncertainty deviation, zone of null deviation and chaotic zone for each data set. Such a determination is not possible on the basis of the Shannon entropy alone as a measure for the MAR uncertainty of QCs, i.e., when they are viewed as extensive systems. Finally, the spatial distributions for the zones of the q-uncertainty deviation (gain or loss in information) of the MAR are derived and lead to iso q-uncertainty deviation maps.

## 1. Introduction

Catchments are viewed as complex, dynamical systems [1] described with a degree of uncertainty [1,2]. This uncertainty is usually quantified by the Shannon entropy when information theory is considered. Entropy has been related to complexity [3], unstructured behaviour and the unpredictability of hydrological systems [4]. Since the complexity reduction of a system is translated into information gain [5], the two concepts could be used interchangeably, depending on the context. There is no doubt about the popularity of the Shannon entropy in diverse fields [6], in particular, hydrology and water resources [7,8,9]. Despite its wide spread applications, the Shannon entropy has a limitation to be confined to extensive systems [4,6]. These systems obey the Boltzmann–Gibbs (B-G) function [10] and are usually assumed to be in equilibrium from the thermodynamics point of view. Thermodynamics assumes that these systems will depend on their initial state whenever they undergo transformation. This could be applicable mostly to ideal systems. However, the majority of the real systems, e.g., hydrological systems such as catchments, do not follow the thermodynamic equilibrium law. To some degree, they could be approached as metastable systems; however, they are more dynamic in nature. The adaptive complexity nature characterises hydrological systems [1], at sub-catchment, catchment and basin level.

Tsallis entropy was introduced to quantify the uncertainty associated with complex dynamic systems, which are not necessarily at the thermodynamic equilibrium since Boltzmann–Gibbs–Shannon (B-G-S) entropy cannot strictly speaking be used for non-equilibrium systems [6]. As many other entropy functions, e.g., the Renyi entropy and the Tsallis entropy, this function takes its origin from the thermodynamics of nonextensive systems and can be maximised by q-exponential family members [10]. The degree of nonextensivity [11] or q-information parameter/index introduced by Tsallis could yield several values of uncertainty for the same set of hydrological records, whereas the Shannon entropy is restricted to a single value for the same data set. For that, in this study it is argued that the q-information parameter could be viewed as a means of describing the dynamic level associated with hydrological systems. Hence, the Tsallis entropy is considered as one of the generalised formulations of entropy [11,12], i.e., the genearalised B-G-S entropy [13]. The Tsallis entropy makes use directly of the q-logarithm function, which is an inverse of the generalised q-exponential function [13].

Despite its spread and use in physics and many other fields [6], its application in hydrology and water resources, specifically, catchment hydrology is limited, e.g. [4,14]. In particular, the review of Tsallis entropy applications for modeling in water engineering that include systems related to frequency distributions, network evaluation and design, reliability of water distribution networks and problems requiring coupling entropy with other theories was recently presented [4]. Thus, the use of the nonextensive approach seems adequate to analyse the complex mechanism of events such as an earthquake [15] as a single event. Hence, the level of complexity increases in catchment where multiple events do occur.

Nonextensive systems are among systems that experience long-range spatial correlations or long-range memory effects [15], unlike extensive systems which are characterised by short range interactions [16]. For instance, runoff resulting mainly from precipitation is characterised by a memory length or response when compared with rainfall occurrence. This response alone exhibits complexity and depends on many hydrological characteristics of the catchment and other variables such as evaporation, soil moisture, etc. The memory length shows usually the delay between the occurrence of precipitation and runoff. Runoff as a hydrological contributor to the catchment yield in water resources has been instrumental for planning and development of water resource systems.

For water resource systems, the quantification of entropy has evolved on variables such as rainfall, streamflow, or runoff and so forth [8,9]. Since these variables are associated with complexity or uncertainty, the level of complexity is even of higher order for hydrological systems such as catchments. The limitation of most entropic calculations (either the Shannon entropy or the Tsallis entropy) in many fields is that they are defined in terms of specific variables of a system, which may not fully capture the core functioning of the entire system. The selection of variables could be partly attributed to the incompleteness of information about the system or the low level of understanding of the system. However, the above-mentioned two expressions of entropy give an acceptable assessment of the uncertainty, as far as the field of hydrology and water resources is concerned.

The present article explores primarily the multiple value entropy from the Tsallis q-information parameter for assessing catchment uncertainty associated with mean annual runoff (MAR) rather than a single value entropy, as in the case of the Shannon entropy. This study is limited only to QCs in different tertiary catchments (TC) of the Middle Vaal. Firstly, to account for the information/uncertainty associated with the MAR of QCs within TCs, the values of the q-information parameter are used for QCs of each TC in the data set. The relative uncertainty is used as a means for comparing QCs in different TCs. Secondly, the q-uncertainty deviation concept is clearly formulated within the hydrological context and assessed to determine the level of metastability of QCs with respect to their thermodynamic equilibrium state. Yet, it is acknowledged that q was stated as a parameter measuring how far a system has deviated from the B-G relationship in the specific case of electromagnetic space plasma environment [16]. Finally, a spatial distribution of q-uncertainty deviation regions for the MAR was also generated for the Middle Vaal catchment, i.e., regions of positive, negative and null deviations could be established. In this way, iso q-information deviation regions could be defined with similar behavioural characteristics that are positive, negative and null. The baseline for the q-information deviation is the Shannon entropy. The chaotic behaviour of these zones is also analysed and discussed. South African hydrology gives a nested approach for catchment subdivision, i.e. QCs form a TC and constitute the foundational entity for the MAR estimation in the respective TC. Hence, TCs with hydrological similarity form a secondary catchment and a group of secondary catchments yields a primary catchment. Any QC (e.g. C41A) is represented with four characters, i.e. the first character relates to the primary basin and the last letter is unique to the given QC. The intermediate letters relate to the secondary and tertiary catchments respectively, in which this specific QC falls. 

The Lower Vaal together with the Upper Vaal and Middle Vaal basins form the current Vaal Management Area (VMA). Like [5], surface water resources (WR90) of the South Africa 1990 (WR90), 2005 (WR2005) and 2012 (WR2012) data sets were used for the MAR, specifically for the Middle Vaal. In the history of South African hydrology, these data sets have been comprehensively published in 1994, 2011 and 2016, respectively. They are freely available on the Water Research Commission (WRC) of South Africa website and they were commissioned by WRC in 1990, 2005 and 2012, respectively. They are a good data source for hydrologists and water resource practitioners and constitute the basis for water resource development and management in South Africa. Hence, as more data become available, new studies are commissioned. Water resource systems in particular are characterised by the MAR.

In the data sets, WR90, WR2005 and WR2012, the Middle Vaal basin is still categorised as one of the 19 WMAs of South Africa, and these data sets are currently used in water related studies in South Africa. The new configuration of WMA yielded 9 WMAs and does not change the validity of the data records for each QC or TC published in the data sets. Moreover, no data has been published referring to the new configuration. Hence, the new configuration does not impact the hydrological records in the Middle Vaal basin at all levels, i.e., primary, secondary, tertiary and quaternary catchments [5]. In this study, the word “basin” is used more than “WMA”, for the Middle Vaal. Often the prefix “meta” before stability will be omitted. Hence, metastability and stability can be used interchangeably, as far as catchments are concerned. “Marginal entropy”, “marginal entropy index” and “entropy” will be used interchangeably. The q-information index, q-information parameter, q-uncertainty index and q-uncertainty parameter will mean the same. Concepts of q-information deviation, q-uncertainty deviation or q-complexity deviation will be used to mean the same concept, since information, uncertainty and complexity are all related. Pseudo and quasi-prefixes for thermodynamic equilibrium will be used interchangeably and may be also omitted in most cases.

The rest of the study is structured in the following way: Section 2 presents very briefly the Middle Vaal catchment and data sets used, nonetheless, further details can be found in [5]. Section 3 firstly gives the methodological approach to determine the q-information deviation curves that depict the dynamical behavior of QCs for each data set. Special conditions of the convergence point of q-information deviation curves for specific QCs are also assessed. Secondly, the spatial distribution of q-information deviation of for each data set is defined and yields the iso-q information deviation map derived for the different QCs in each data set. Section 4 presents the results and discussion derived from the application of the methodological approach. Section 5 deals with conclusions and suggestions of the study.

## 2. Study Area and Data Availability

**Figure 1:**
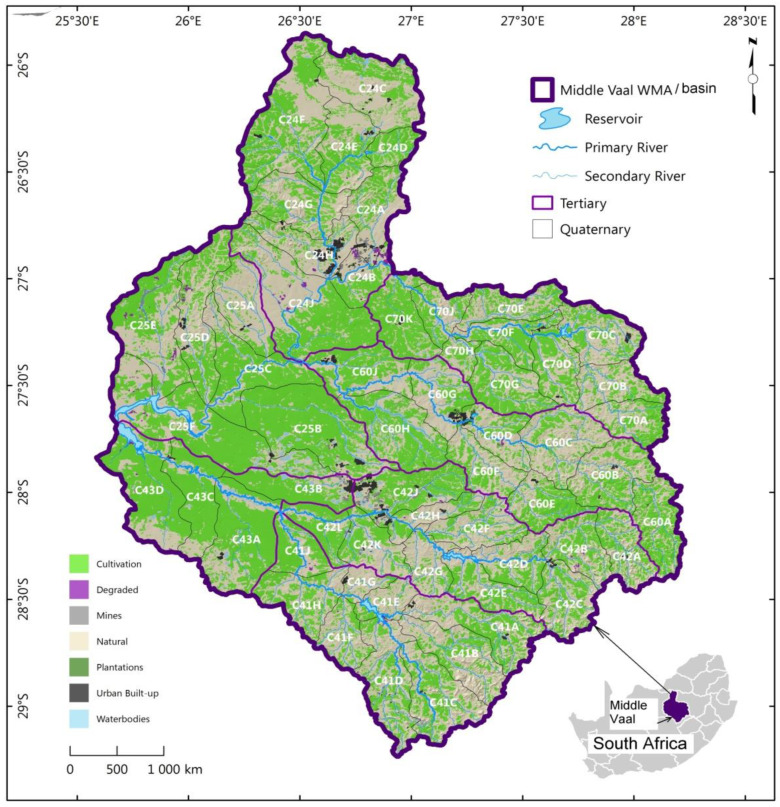
Quaternary catchments in their respective tertiary catchments of the Middle Vaal basin of the Republic of South Africa shown at the bottom right; WMA: water management area.

This section has been essentially derived from the previous study [5]. For more details, the reader is referred to this study. The Vaal region is situated in Gauteng Province and is comprised of three basins which are the Middle Vaal, Upper and Lower basins. This province is the most economically developed from its contribution to the gross development product (GDP) of South Africa. Climatic conditions, demographic and economic features and water resources are not the same in all three basins. Nonetheless, the interest of this study is on the Middle Vaal basin, which interacts geographically with the West Crocodile and Marico and the Upper Orange basins. The geographical location of the Middle Vaal is depicted in Figure 1. Unlike in the previous study, where the African map was also associated, this study is only restricted to a map of South Africa, where the focus is on Middle Vaal WMA. The Middle Vaal is divided into three areas, the Rhenoster-Vals formed by TCs C24 and C25, the Middle Vaal sub-area comprising C60 and C70 and the Sand-Vet that is made by C41, C42 and C43.

Grassland with a flat relief and dolomite soil dominate the Middle Vaal. The exploitation of gold mines, agriculture and trade shows the economic dominance in this basin. The Middle Vaal falls in a semi-arid country, which has approximately 400 mm as the mean annual precipitation (MAP). This value is below the world mean annual precipitation. The excessive temperatures in summer from October to February yield a huge loss of rainfall in most parts of South Africa. This situation causes the mean annual evaporation (MAE) to be greater than the MAP, as presented in Table 1. This situation led South Africa in the past to build more water storage capacities, in particular, hydraulic structures such as dams and to establish water transfer schemes among basins in a way to balance water demand and supply [5]. Table 1 has been extracted from the previous study and summarizes data from TCs that include the different QCs. For more details on TCs and QCs, the reader can use the WRC publications, i.e., WR90, WR2005 and WR2012, which include data from 1920 to 1989, 1920 to 2004 and 1920 to 2012, respectively. Hence, the WR2012 data set is so far the latest officially published data set. The values in Table 1 are based on this latest publication. Differences in catchments characteristics, such as geology, soil texture, topography and vegetation contribute to differences in the mean annual evaporation (MAE), mean annual runoff (MAR) and mean annual precipitation (MAP) in the TCs [5].

## 3. Methods

In the following, the Tsallis entropy is mainly explored as a measure of uncertainty/information for catchments.

### 3.1. Relative Tsallis Entropy

Similar to the Shannon marginal entropy index [5], the Tsallis marginal entropy index can be defined below.

In its discrete form, the Tsallis marginal entropy index written as $T{\left(Y\right)}_{i,q}$ of a hydrological variable *Y* (e.g., runoff) in the *i*-th TC catchment (*i* = 1, …, *p*) is determined by Equation (1):

(1) $$T{\left(Y\right)}_{i,q}=\overset{\phi }{\underset{j=1}{\sum }}T\left(y_{j}\right),q$$

where ϕ is the number of QCs in the *i*-th TC catchment of a basin, q is the information parameter and $T\left(y_{j}\right),q$ is the Tsallis uncertainty contribution of the event *y_j_* (*j* = 1, 2, …, ϕ) describing the variable Y. The value *y_j_* can be MAR for a specific *j*-th QC in a given TC of a basin, i.e., the Middle Vaal. The MAR of a TC is made from the MAR contribution from each QC.

*If*$\delta_{j}=\;\frac{y_{j}}{Y_{\phi }}$*such that*$Y_{\phi }=\sum_{j=1}^{\phi }y_{j}$, the Tsallis marginal entropy (with q-information parameter) of the *i*-th TC catchment is determined by Equation (2) below:

(2) $$T{\left(Y\right)}_{i,q}=-\frac{1}{q-1}\sum_{j=0}^{\phi }(\delta_{j}{}^{q}-\delta_{j}).$$

Equation (2) emanates from the generalised B-G-S entropy for nonextensive statistical mechanics [13].

Hence, the uncertainty contribution of the *j*-th QC in the Tsallis entropy of the *i*-th TC is given by Equation (3)

(3) $$T{\left(y_{j}\right)}_{,q}=-\frac{1}{q-1}(\delta_{j}{}^{q}-\delta_{j}).$$

It should be noted that Equations (1) and (2) satisfy the following condition:

(4) $$\sum_{j=1}^{\phi }\delta_{j}=1.$$

The determination of the q-information parameter is not straightforward. This can be possible in specific situations, for example when the microscopic probabilistic/dynamical details about a system are completely known, and this is very rare, hence, first principles (i.e., from dynamics and theory of probabilities) can be used to calculate q a priori [6,13]. In other cases, many details of the system influence the q-values and the determination of q for all existing systems is still at an early stage [6]. In many instances, the system details are not known a priori [6], and determining q-values is not an easy task [17]. Hence, for physical, biological and ecological systems, and so forth, there is no general methodology to determine the q-values [18]. For example, in the complexity assessment of new biological species discovery rates using entropy, low order positive q-values not exceeding 4 were tried and the parameter q determined the measure’s sensitivity to species relative abundances [19]. In the estimation of the biodiversity of a community using entropy concept, the nonextensive parameter was chosen between 0 and 2 [20]. A selection of three q-values, i.e., 0.1, 0.5 and 2.5 were chosen to check the sensitivity of entropy for land use/cover change [21]. Multiple values of q were selected to demonstrate entropy applications for modelling in water engineering [4] and for one-dimensional movement of moisture in unsaturated soils [22]. The behavior of catchments is very dynamic and nonlinear and so far not well known, hence, several values of q will be tried to evaluate the effect on entropy in the present study.

Positive values of the q-information parameter display the nonextensivity properties [12] of hydrological systems [4]. Since catchments could be assumed in a metastable state rather than thermodynamic equilibrium state, they could be considered as nonextensive hydrological systems. In fact, a catchment that has undergone development (e.g., land use/change, etc.) would unlikely depend on/or return to its original/virgin conditions.

The Tsallis maximum entropy in the *i*-th TC noted as $T_{i,q\;Max}$ is given by Equation (5)

(5) $$T_{i,q\;Max}=-\frac{1-\phi^{1-q}}{q-1}.$$

Equation (5) holds subject to the constraints in Equation (4).

$T_{i,q\;Max}$ corresponds to the equi-probable outcomes of the hydrological variable for given values of q and for any *j*-th QC in the *i*-th TC. The distribution of the hydrological variable for entropy values closer to the minimum value is compact, as opposed to values closer to $T_{i,Max}$ that show an evenly dispersed or spread distribution.

The relative uncertainty contribution of *j*-th QC could be used to standardise the comparison between the uncertainty contribution of the *j*-th QC and the maximum entropy in the *i*-th TC [5], and is defined by Equation (6) as the ratio between Equations (3) and (5):

(6) $$R_{j,q}=\frac{T_{j,q}}{T_{i,q\;Max}}.$$

Higher ratios are characterised by higher uncertainty contributions of the *j*-th hydrological sub-system (i.e., QC) to the *i*-th hydrological system (i.e., TC). Systems with entropy tending to maximum uncertainty have been shown to have a higher spread spatial distribution of the hydrological variable [5]. Conversely, systems with low uncertainty seem to concentrate much of their hydrological variable in few QCs, as opposed to the rest of QCs. There is always a transformational process between data sets that could be characterised by the change in entropy. Generally, this transformation can be translated into gain or loss of uncertainty between data sets.

Relative entropy can be related to information increase, or decrease. Since the relative entropy enables to define the complexity of hydrological systems [5], it could be used as a means for comparing QCs in the different TCs of a given data set. The mono-variable approach in entropy assessment can be a limiting factor in determining the complexity of hydrological systems [5]. In principle, the definition of variables depends on the system as well as on the scale of observation [23].

### 3.2. Concept of Q-Information/Uncertainty Deviation

In this study, the difference between the Tsallis entropy and the Shannon entropy is referred to as the q-uncertainty deviation. The values of uncertainty should be expressed in relative terms. Given an *i*-th TC, the q-uncertainty deviation shows the proportion by which the Tsallis uncertainty contribution of each QC has decreased or increased with respect to its Shannon uncertainty. The Shannon entropy is considered to be the reference, since it shows the ideal case of a system associated with the B-G relationship. Hence, mathematically, the q-information deviation is defined by Equation (7), as given below.

(7) $$D{\left(y_{j}\right)}_{,q}=T{\left(y_{j}\right)}_{,q}-S\left(y_{j}\right)\;\;\;\;\;q\;>\;0$$

where $S\left(y_{j}\right)$ is defined as the Shannon uncertainty.

At the limit when q ―› 1, the following expression, i.e., Equation (8) is obtained

(8) $$D{\left(y_{j}\right)}_{,q}=0.$$

The above equation is justified by that fact that the Tsallis uncertainty and the Shannon uncertainty are equivalent at the limit [13,15]. Hence, in this case, the Shannon entropy can be a good approximation for metastable nonextensive systems that, in reality, could be dynamic complex systems such as catchments. Systems in the state of quasi equilibrium could be characterised by the nonextensivity parameter q that quantifies the scale of spatial interactions for q~1 [15]. When $D{\left(y_{j}\right)}_{,q}>0$, hydrological systems display a positive behaviour (increase in uncertainty) with respect to the thermodynamically equilibrium behaviour. Otherwise hydrological systems tend to behave negatively, since these systems have decreased in uncertainty, as compared to thermodynamic equilibrium.

Complex systems which are in an open spatial nonequilibrium state are characterised by a degree of nonextensivity [24]. These systems can still maintain a status far from equilibrium through self-organisation and are the domain of both ecological and hydrological transformations [1]; catchments can be a good illustration of this kind of systems.

The q-information deviation could be an indicator for comparing catchments to their pseudo equilibrium state. The pseudo equilibrium state of hydrological systems is defined by the theoretic entropy, i.e., the Shannon entropy. Hence, the q-uncertainty deviation enables to assess whether a catchment could be close to its extensive state or departs from it positively or negatively. It is reiterated that catchments exist in a state of pseudo equilibrium, which is different from the equilibrium state of the virgin conditions. Usually, the pseudo equilibrium state of a system is referred to as a metastable state. For instance, terms such as metastability, dynamic equilibrium, homeostasis or stationary state, hence stability, are used as a nonthermodynamic state of ecological systems [25,26] that form part of real systems.

#### 3.2.1. Q-Uncertainty/Information Deviation Curves

High (Low) complexity is characterised by a high (low) Tsallis entropy value and vice-versa [24]. These are curves defined by the complexity of each catchment versus the different values of the q-information parameter based on the Tsallis entropy. Since the nonextensive q-information parameter enables to quantify the complex evolution of a system, q-uncertainty deviation curves could show the dynamic evolution of a catchment within the same set of data. Such an evolution would have not been detected with the Shannon entropy, which is suitable for systems in the state of quasi equilibrium. The increase of q parameter values, showing that the system state goes away from equilibrium [15], could be associated with the spatial dynamic evolution of the catchment.

In the current study, the dynamic evolution was described by the positive, negative or indifferent behaviour of the catchment with respect to the thermodynamic equilibrium behaviour. In this case, the behaviour is translated into deviation. The Shannon entropy could be seen as a single value belonging to this type of curve. An assessment of the trend of the iso-uncertainty deviation curves was assessed and could show how well they departed from the Shannon entropy.

#### 3.2.2. Existence of Convergence/Intersection Point of Q-Uncertainty Deviation Curves

Two or more q-uncertainty deviation curves converge if only if there is a point defining the same level of complexity. Hence, for any *i*-th and *j*-th QC, at least one value of q satisfies the following condition

(9) $$T{\left(y_{i}\right)}_{,q}=\;T{\left(y_{j}\right)}_{,q}.$$

#### 3.2.3. Identical Q-Uncertainty Deviation Curves

These are q-information deviation curves that characterise two or more QCs (with *i* ≠ *j*) such that the following condition is satisfied for all q-values,

(10) $$T{\left(y_{i}\right)}_{,q}=\;T{\left(y_{j}\right)}_{,q}.$$

In other words, two or more QCs that display approximately the same values of the Tsallis entropy everywhere, i.e., having the same values of q-information deviation and constitute identical q-information deviation curves. These QCs have similar complexity levels. Considering such curves in the complexity assessment of the respective TC would be a duplication of information or redundancy that is related to repetitive information. The knowledge of uncertainty in one of the curves defines the information content in the rest of identical q-information deviation curves.

### 3.3. Defining Iso Q-Uncertainty/Information Deviation Maps

Extensive QCs are assumed to follow a quasi B-G-S entropy approach. For the purpose of this study, iso-uncertainty deviation defines the same sign of q-uncertainty deviation for different hydrological systems. Referring to the previous section, the Tsallis entropy may or may not prevail over the Shannon entropy for specific QCs in a given TC. Hence, three categories of iso-q uncertainty zones will be defined for catchments:

- Zone 1: $D{\left(y_{j}\right)}_{,q}>0$; nonextensive metastable properties prevail and show an increase in uncertainty from the Shannon entropy
- Zone 2: ${\left(y_{j}\right)}_{,q}<0$; nonextensive metastable properties prevail and display a decrease in uncertainty from the Shannon entropy
- Zone 3: $\left|D{\left(y_{j}\right)}_{,q}\right|\leq \beta$ for all q-values and 0 ≤ β <<< 1; nonextensive properties prevail with QCs displaying the level of stability from the extensive state. Zone 3 is a non-chaotic zone during hydrological transformations that occurred in the data sets.

The spatial representation of these three zones determined the q iso-information deviation maps for WR90, WR2005 and WR2012. Hence, the zones of the same sign could be determined and analysed. There is no universal rule to define entropic zone values; hence, they are usually determined arbitrarily, as far as entropy related studies are concerned [5]. The maps in this case could show the level of relative stability (metastability) of the MAR of QCs over TCs, when considering the MAR distribution in the different QCs.

The specific case of QC instability will be identified such that for a given QC in a TC, the iso q-uncertainty deviation should satisfy the uncertainty difference, as given in Equation (11) below:

(11) $$\left|D{\left(y_{j}\right)}_{,q}\right|>\beta$$

where β is a small positive threshold value.

Hence, in the case of the above equation, the hydrological system is a chaotic zone and departs significantly from the metastable conditions, i.e., when the q-deformation uncertainty/complexity difference exceeds the threshold. The QC displays the level of instability if its relative uncertainty departs by at least β from the extensive state. Referring to the zone of catchment instability, this zone corresponds to the zone of water resources becoming vulnerable, hence, loosing resilience [3]. There is almost no literature to define this threshold, as this approach could be preliminarily the first in its kind for hydrology and water related studies. For that, β was arbitrarily chosen in terms of relative uncertainty difference, for instance β≽0.1. This implies that a QC displays the level of instability if its uncertainty departs by 10% or more from the Shannon entropy.

#### Frequency of Q-Uncertainty Information Zones

For a given data set, let N_+_, N_−_, and N_0_ be the number of behavioural categories or zones, i.e., positive, negative and null q-information deviation respectively. Hence, the total number of behavioural categories for all QCs is given by

N = N_+_ + N_−_ + N_0._ (12)

The frequency of positive q-information deviation zones is

N = [N_+_/N] × 100. (13)

Similarly, the frequencies for negative and null q-information deviations are given respectively by

N_−_ = [N_−_/N] × 100 (14)

N_0_ = [N_0_/N] × 100. (15)

If there is no null q-information deviation zones, Equation (12) becomes

N = N_+_ + N_−._ (16)

Any zone that dominates the iso q-information deviation map will have the highest frequency.

## 4. Results and Discussion

### 4.1. MAR Uncertainty and Q-Information Deviation in the Middle Vaal

**Figure 2:**
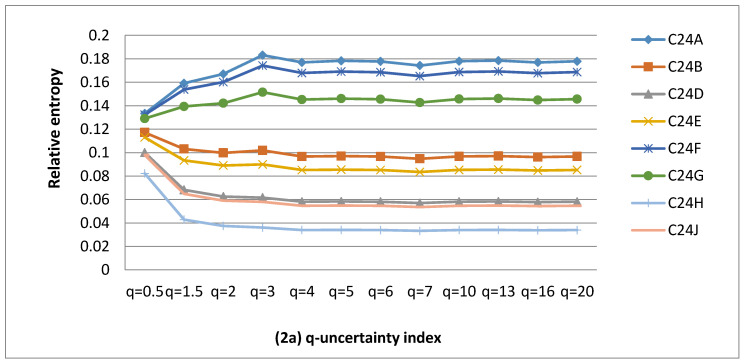

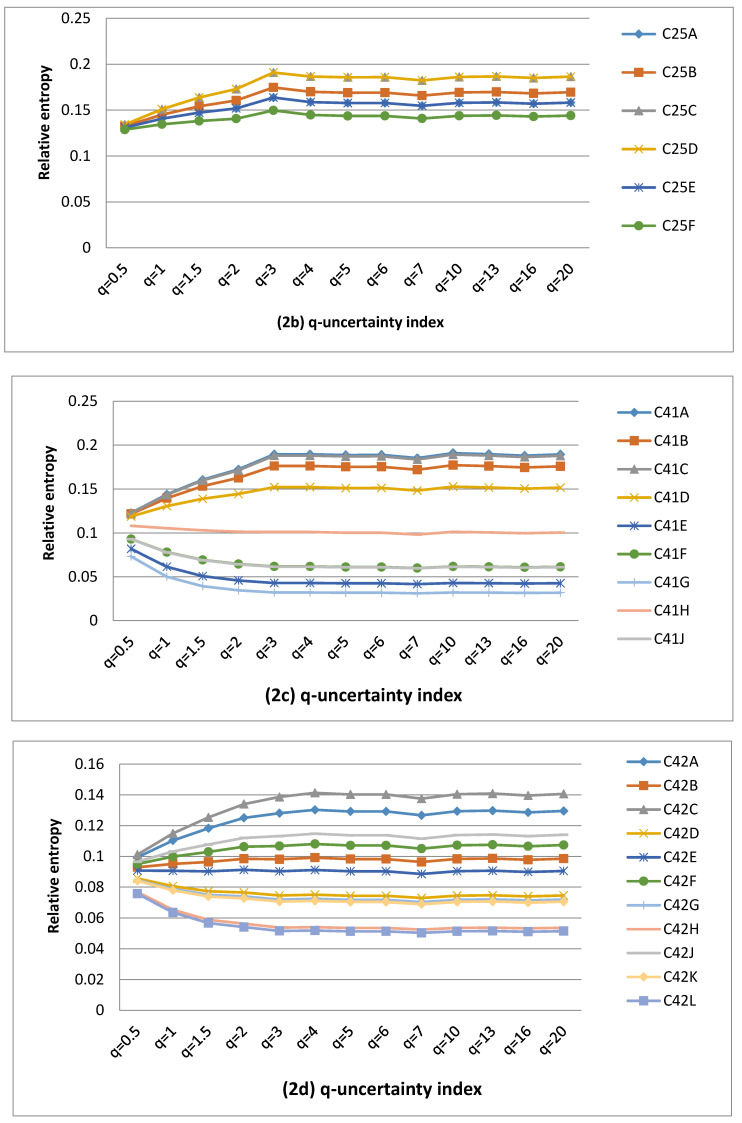

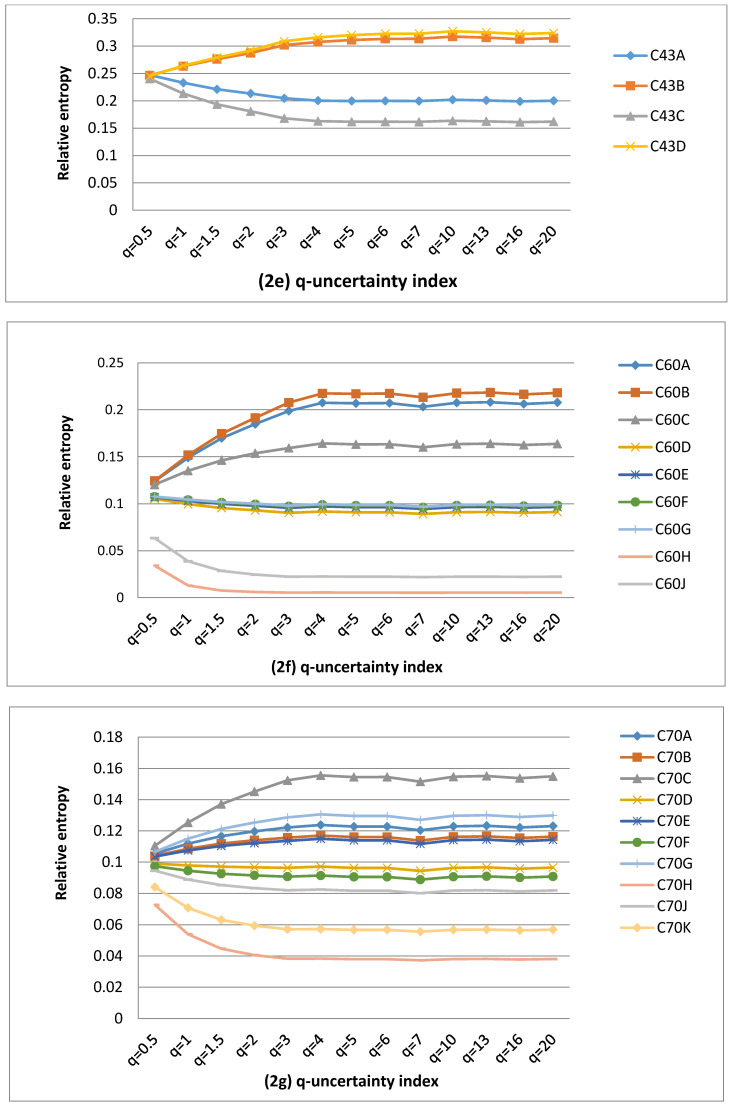
(**a**–**g**) Q-information deviation curves in tertiary catchments C24, C25, C41, C42, C43, C60 and C70 for WR90.

**Figure 3:**
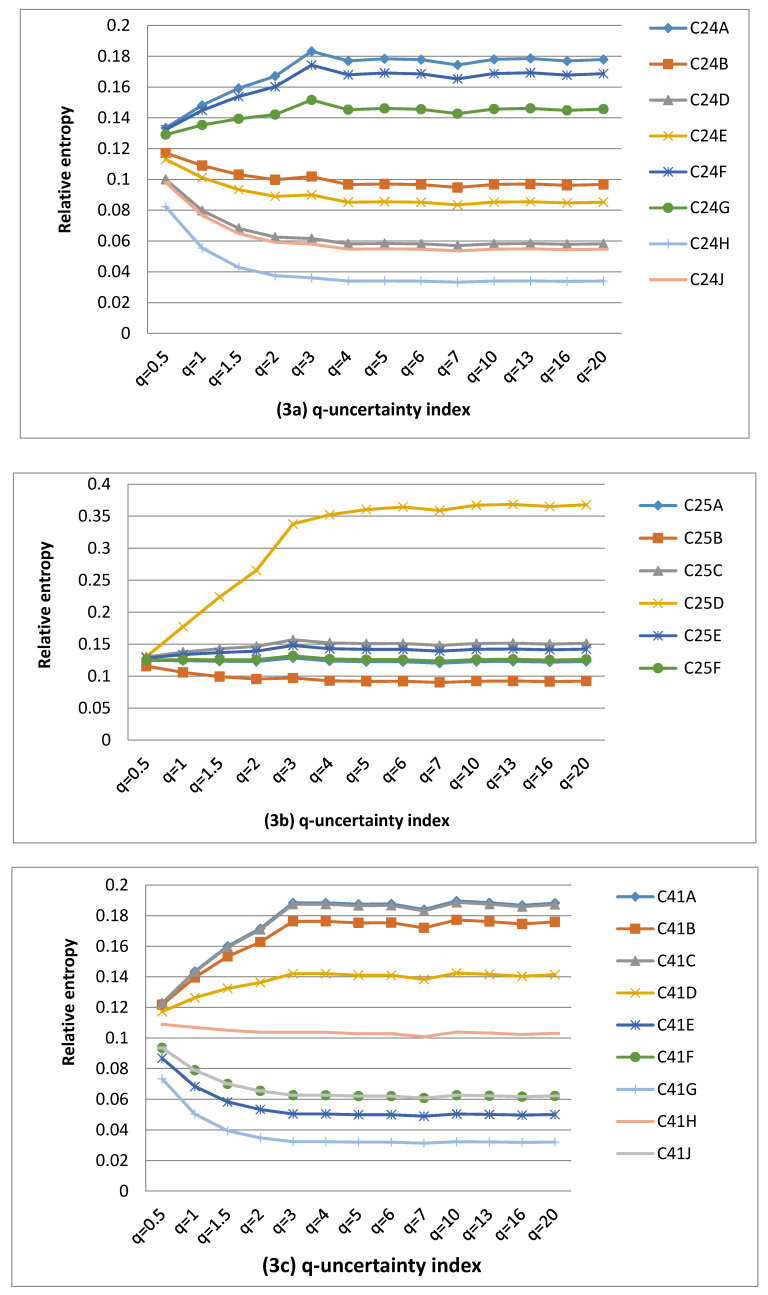

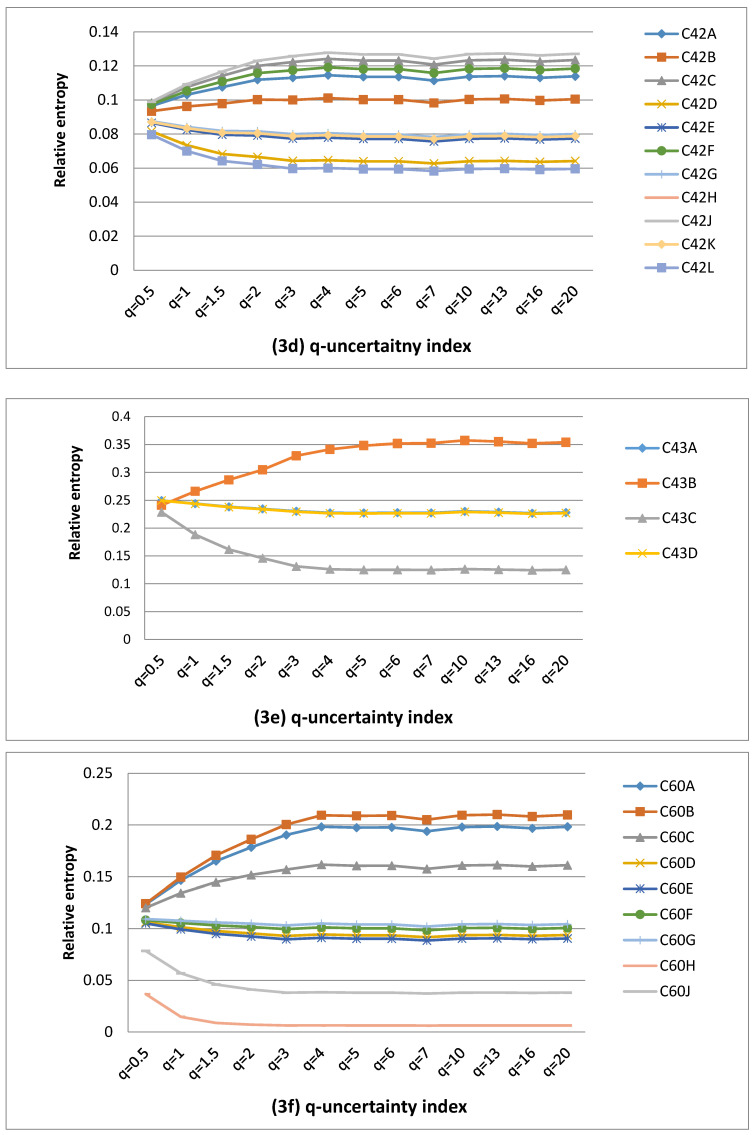

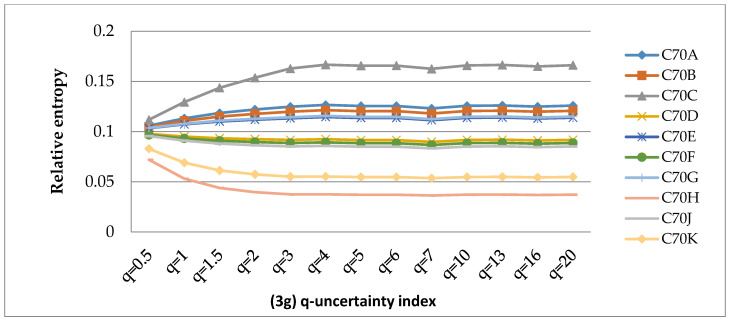
(**a**–**g**) Q-information deviation curves in tertiary catchments C24, C25, C41, C42, C43, C60 and C70 for WR2005.

**Figure 4:**
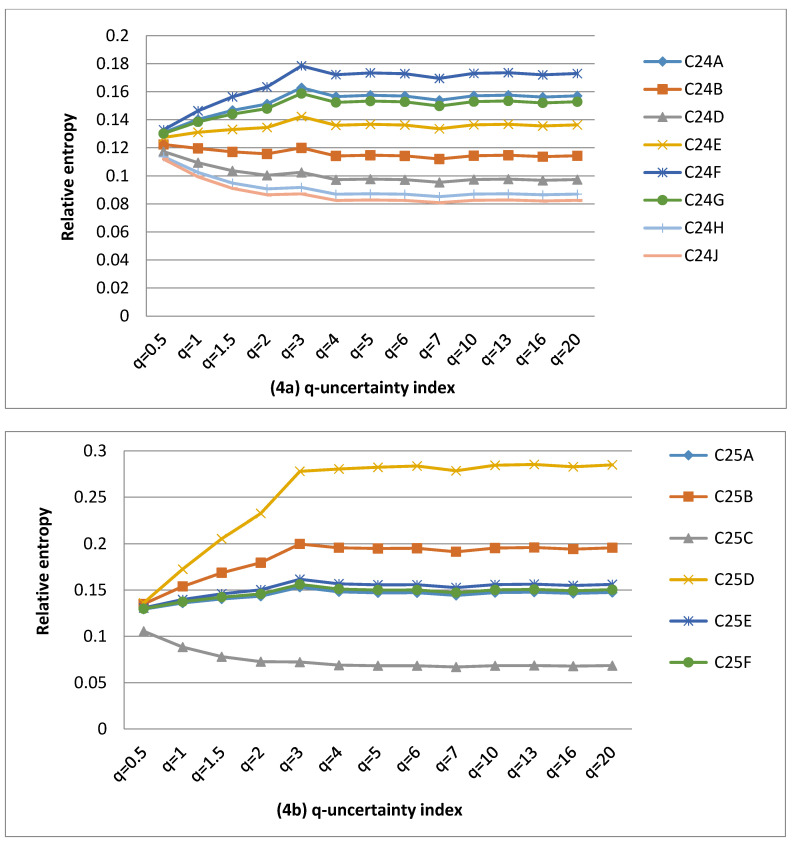

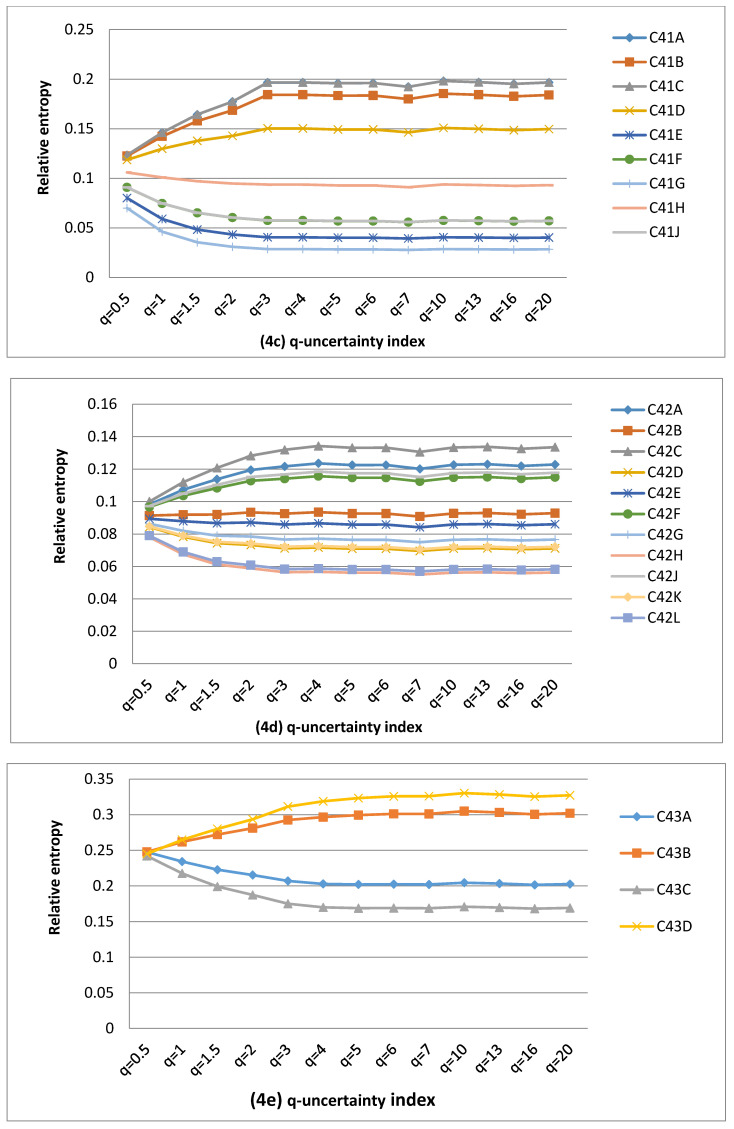

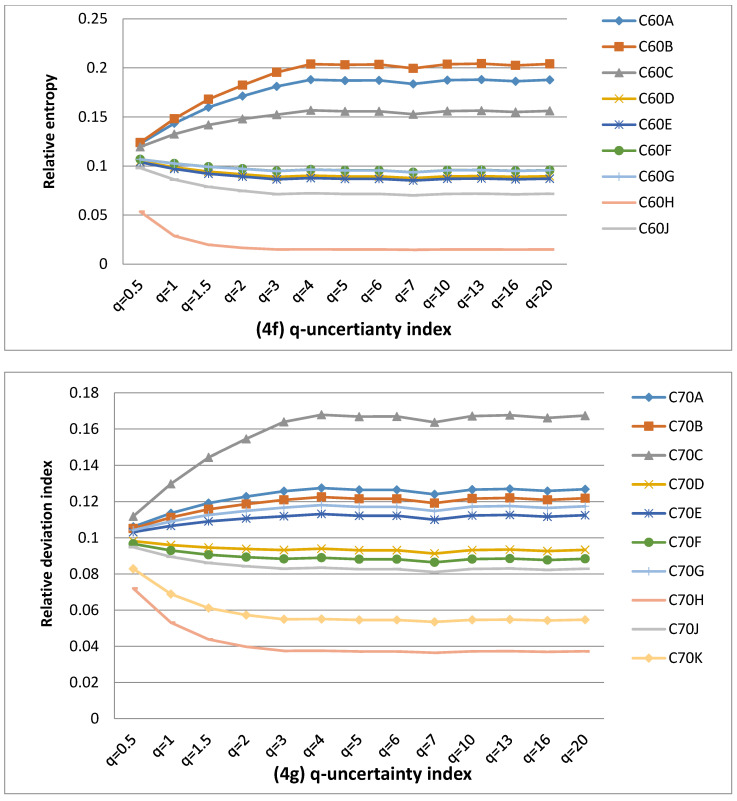
(**a**–**g**) Q-information deviation curves in tertiary catchments C24, C25, C41, C42, C43, C60 and C70 for WR2012.

The calculations of uncertainty values associated with MAR between 1990 and 2012 were carried out and plotted in Figure 2, Figure 3 and Figure 4. Catchment complexity was restricted to MAR uncertainty in the Middle Vaal basin. Different values were considered for q-information parameter for QCs to derive the q-information deviation values. The summary of results was given in Table 2, Table 3 and Table 4. These tables showed the behavioural characteristics of q-information deviation curves associated with MAR for the different data sets, in the Middle Vaal, i.e., number of entropy convergence points, null information deviation, positive information deviation, negative information deviation, identical information deviation and chaotic zones (QCs) in the different TCs.

The results were analysed and discussed for QCs in their respective TCs considering all data sets, i.e., WR90, WR2005 and WR2012. For each data set, Figure 2, Figure 3 and Figure 4 revealed that the relative uncertainty as a measure of catchment complexity [9] was approximately bound between an upper and a lower value when the q-information parameter varied. For the different QCs, generally, the abovementioned figures revealed that the catchment complexity increased until a certain value of q-information parameter and remained approximately constant beyond that value. In specific TCs, it was revealed that the uncertainty remained more or less the same with respect to the q–information parameter. In general, the MAR uncertainty was not further sensitive to the q-value values around 5.

**Figure 5:**
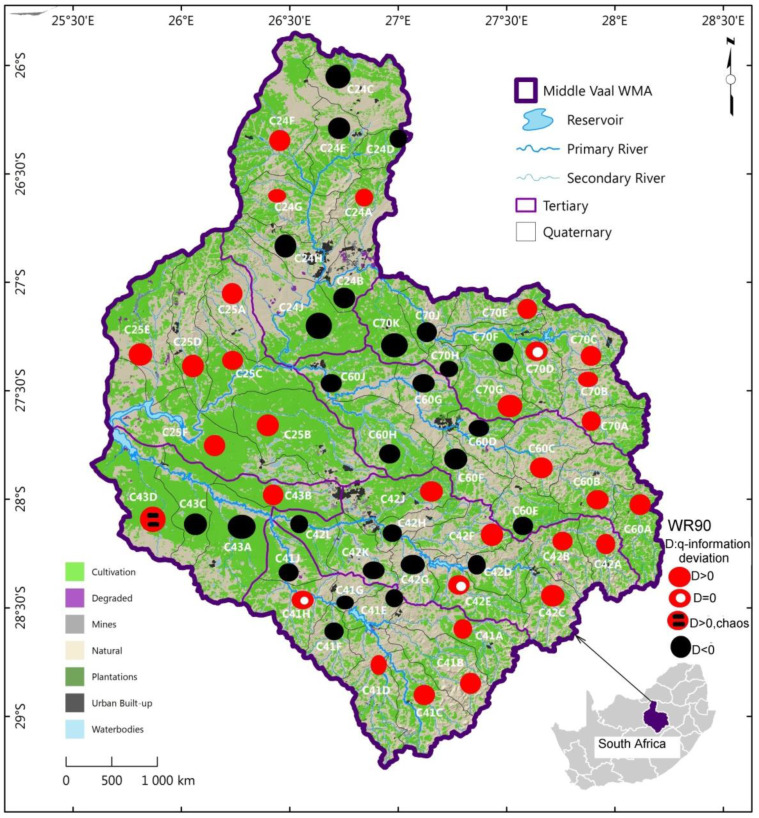
Spatial distribution of q-information deviation for QCs of TCs in the Middle Vaal basin; for data set WR90; WMA: water management area QC: quaternary catchment, TC: tertiary catchment.

**Figure 6:**
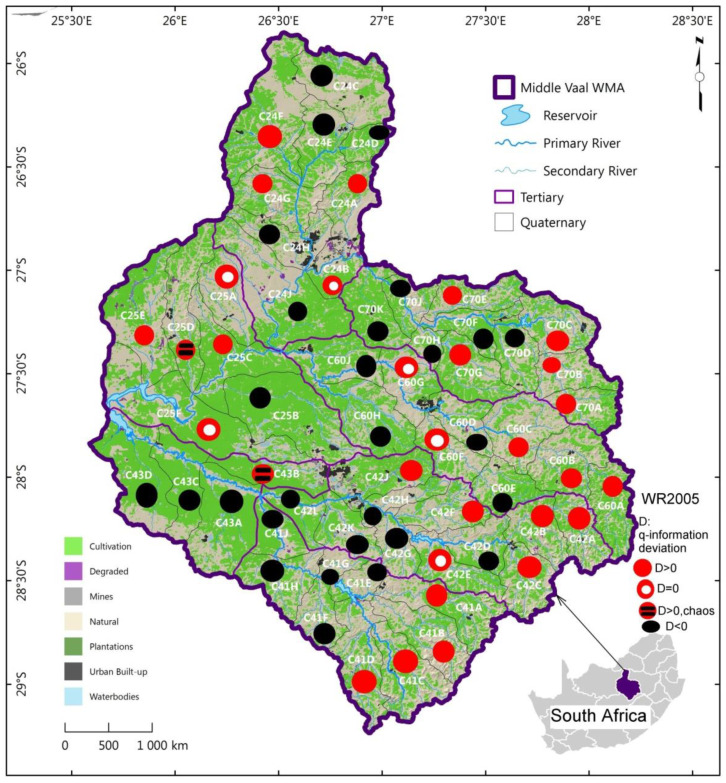
Spatial distribution of q-information deviation for QCs of TCs in the Middle Vaal basin; for data set WR2005; WMA: water management area QC: quaternary catchment, TC: tertiary catchment.

**Figure 7:**
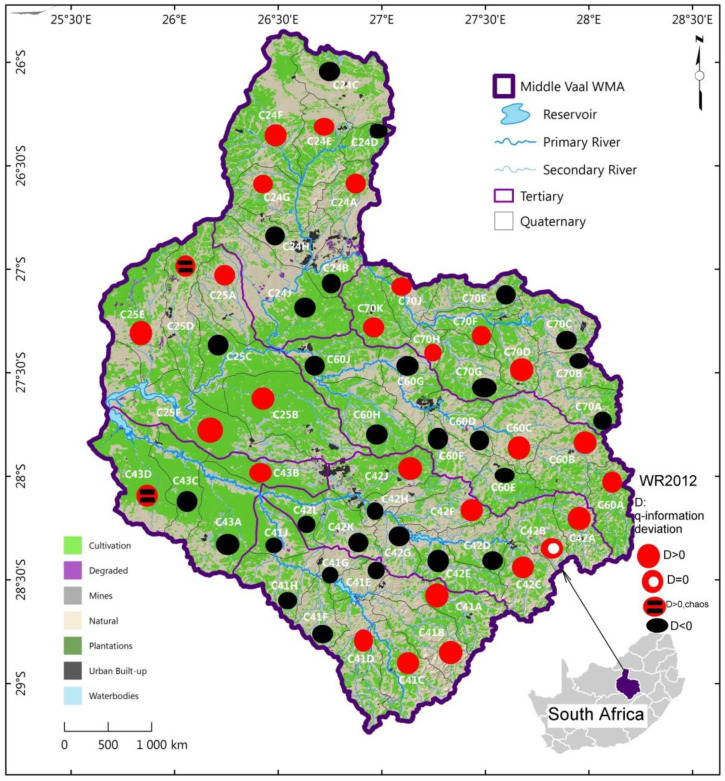
Spatial distribution of q-information deviation for QCs of TCs in the Middle Vaal basin; for data set WR2012; WMA: water management area QC: quaternary catchment, TC: tertiary catchment.

The spatial distribution of the three categories of q-information deviation, i.e., positive, negative and null associated with the catchment yield (MAR) were displayed in Figure 5, Figure 6 and Figure 7 for the three data sets respectively, as presented in Section 4.2.

#### 4.1.1. Influence of Q-Information Parameter on MAR Uncertainty for WR90

Figure 2a–g revealed that both positive and negative q-information deviation values occurred for WR90. As presented in Table 2, the proportion of positive q-information deviation regions were dominant (i.e., 48%) as opposed to 47% of negative q-information deviation regions; however, the difference between these two category occurrences was small. That is to say that, with respect to the Shannon entropy, the majority of QCs displayed an increasing trend of complexity as compared to those characterised by decreasing complexity. This could mean that the majority of QCs were in the metastability state, departing positively from the equilibrium state; however, both positive and negative q-information deviation regions displayed nonextensive properties. This could be in line with the Tsallis entropy formalism for nonextensive systems [11,13], which are nonideal systems such as catchments. The slight dominance of positive q-information deviation catchments over negative ones could signal that more QCs are in the state of relative higher uncertainty.

Hence, most QCs could be described better by the Tsallis entropy as opposed to the Shannon entropy, except C41H, C42E and C70D QCs which had null q-information deviation and represented only 5% of the total QCs in the basin. The MAR information content for these catchments could be described specifically by the B-G-S entropy and could be believed to display extensive properties in their metastable state. Hence, they could be assumed in pseudo thermodynamic equilibrium conditions.

C43D was revealed to be in a chaotic zone; hence, its water resources resilience/sustainability could be compromised, since the q-information deviation was above the threshold. However, the water resources in this QC represent not more than 2% in the Middle Vaal River system and are subject to vulnerability. This could imply that the rest of QCs could outweigh the negative impact (water vulnerability) of C43D on the overall basin in terms of water resources usage. This could support the current practice of transferring water between catchments to resolve the water deficit in some parts of South Africa.

TCs C25 and C43 scored the highest and lowest numbers of QCs occurrences, respectively, in terms of positive q-information deviation. This suggests that C25 could be characterised by high MAR complexity as opposed to C43. It would require more information to reduce the uncertainty for C25 than C43 to reach equilibrium conditions. For the negative q-information deviation, the highest and lowest numbers of QCs occurred in TCs C60 and C43, respectively. This result could mean that a relatively low MAR complexity dominated C60, as compared with C43. Hence, C43 scored the lowest occurrences for both q-information deviation categories. All TCs had QCs displaying both negative and positive q-information deviation, except C25, where there was no occurrence of negative q-deviation information. When considering the WR90 data set, there is only one TC where QCs required strictly gain of information.

There is existence of convergence points (in total 14) of relative entropy values. Such points cannot be defined by the Shannon entropy, but by the Tsallis entropy, which is translated into a multistage entropy configuration determined from the q-values for the same data set. During this configuration or entropic evolution, it is believed that there could be a unique state of common uncertainty to specific QCs in a given TC.

A total of 14 curves displaying identical q-information deviation were identified for WR90 data set. These curves suggested that there was duplication of information/uncertainty in specific QCs. For instance, defining MAR information in one QC was sufficient to define information content in all QCs identical to that QC. For this specific data set, this number was found to be the same as the number of entropy convergence points for q-information deviation curves.

#### 4.1.2. Influence of Q-Information Deviation on MAR uncertainty for WR2005

Like in WR90, Figure 3a–g revealed that both positive and negative deviation values occurred in WR2005, however, the proportion of the negative q-information deviation areas was dominant (i.e., 48%) as opposed to 41% of positive q-information deviation areas. This could mean that most QCs were in the state of metastability, departing negatively from the pseudo equilibrium state, however both positive and negative q-information deviation QCs displayed nonextensive behaviour, based on the Tsallis entropy concept. Similar to WR90, the dynamic evolution of QCs could feature within WR2005. The difference was nearly 7% between positive and negative q-information deviation areas and could be small in relative terms. This could yield a fair q-information deviation category distribution in this basin. The small dominance of negative q-information deviation catchments over positive ones could signal that most QCs were in the state of relative lower uncertainty.

As noticed previously, the MAR information content of the majority of QCs could be computed better using the Tsallis entropy than the Shannon entropy, except C24B, C25A, C25F and C60G QCs, for which uncertainty values remained constant for the different values of q. These four catchments satisfy the B-G-S entropy. C25D and C43B were each found in the chaotic zone. Similar to WR90, these two QCs do not exist in isolation from other catchments of the Middle Vaal; hence, they are part of an integrated management approach of water resources of South Africa. Hence, the vulnerability of these QCs could be far outperformed by the resilience of water resources in other QCs. Nonetheless, these results prove that water managers should closely monitor water resources within the two chaotic zones.

TCs (C42, C70) and C43 scored the highest and lowest numbers of QCs occurrences in terms of positive q-information deviation respectively. This suggested that the requirement for information gain to reduce the level of uncertainty to reach the baseline uncertainty could be more prevalent for C42 and C70 than for C43. However, the highest and lowest numbers of QCs occurred for (C42, C70 and C41) and C25 in terms of negative q-information deviation, respectively. Hence, C42, C70 and C41 would require more loss of information than C25 to reach the baseline uncertainty.

Catchments C24B, C42A, C25F, C42E, C60G and C60F displayed null q-information deviation and constituted 11% of the total QCs. Hence null categories in WR2005 were higher than those in WR90. Hence, the Shannon entropy could be used to evaluate the complexity for these QCs in their metastable state. These catchments would not be affected by the dynamic evolution behaviour determined by q-information parameter, within WR2005. In this instance, the limit of q towards 1 could be justified where the Tsallis information coincides with the Shannon information.

It was noticed that the number of convergence points for the data set WR2005 was almost the same as that of WR90. Temporal hydrological transformation, i.e., between WR2005 and WR90 could not have an influence on the number of unique states of common uncertainty in a specific TC. As compared with WR90, data set WR2005 scored a higher number of identical q-information deviation curves. Hence, the level of duplication/redundancy of MAR information for QCs could be relatively higher in the former data set than the latter.

#### 4.1.3. Influence of Q-Information Deviation on MAR Uncertainty for WR2012

Figure 4a–g revealed that the proportion of negative q-information deviation areas was higher (52%) than the positive (46%) q-information deviation areas. This is similar to WR2005. Referring to the the results from the previous section, it could be said that relatively more QCs are characterised by lower uncertainty.

As in the case of WR90 and WR2005, the QCs in the WR2012 data set could be assumed to follow the Tsallis entropy as opposed to the Shannon entropy, except C42B as the only QC for which the entropy value remained unchanged everywhere. Hence, the Shannon entropy could be applied specifically to this QC, which could be easily managed to recover a quasi-stable state in terms of its water resources. However, the state of other QCs far outweighs this catchment and could influence the overall basin to depart from thermodynamic equilibrium conditions. As for WR2005, it was found that C25D and C43D were the only QCs which displayed unsustainable water resources and were categorised as nonresilient zones, since their q-information deviation values were relatively higher than the defined range in this study. Similar to the previous findings, these QC are not managed in isolation from other QCs of the Middle Vaal, but they are part of an integrated water system. Once again, water resource managers should carefully assess the status of these specific QCs.

The QCs with highest and lowest q-information deviation occurred in (C25 and C70) and C43 respectively, in terms of positive q-information deviation. Once again, C70 and C43 featured here similarly to WR2005. However, (C42, C70) and C25 revealed the highest and lowest number of QCs of negative q-information deviation. C70 appeared to have scored for both categories, the highest occurrences.

It was noticed that the number of convergence points of relative entropy for WR2012 was lower than the two remaining data sets. WR2012 scored the same number of identical q-information deviation curves as WR2005.

#### 4.1.4. Comparison of Q-Information Deviation Results between WR90, WR2005 and WR2012

From one data set to the other, the different categories of q-information deviation did not occur necessarily in the same way in all TCs. Nonetheless, both positive and negative categories appeared fairly in reasonable proportions in all data sets as compared to other categories. In particular, C70 appeared to be the epicentre for both positive and negative occurrences for WR2012, specifically, while this was not the case for the other two data sets. It is reiterated that positive and negative categories corresponded to cases of increase and decrease in uncertainty with the Shannon entropy taken as the baseline. At the time of this study, there were only three data sets that were analysed, as more data sets become available, new trends in these occurrences could be further investigated.

The behaviour of identical q-information deviation curves could suggest that the knowledge of the status of water resources in QCs could be necessary to project the status in other QCs in a specific TC. For instance, for WR90, since C41A and C41C are identical in terms of q-information deviation, the determination of the yield in one of these catchments could be enough to know the yield in the other and vice-versa. Hence, considering both catchments in the estimation of the yield of water resources for C41 could be viewed as redundant. Derived from the Tsallis entropy approach, this study defined q-information deviation redundancy as a duplication of information contained in the MAR from two or more QCs in the same TC.

The same could apply to the QC pair C41F and C41J. For example, the evaluation of complexity associated with the MAR in TC C41 would only necessitate information of seven QCs in place of nine QCs. This could reduce the computational burden. In comparison with WR90 and WR2012, WR2005 depicted a relatively higher number of identical q-information deviation curves. With reference to the Tsallis entropic fundamentalism, this could yield to more QCs displaying approximately similar behaviour in terms of the dynamic change of water resources within the same data set.

Altogether, QCs in the data set WR2005 could be believed to have more redundant information than WR90 and W2012. The hydrological change in the Middle Vaal between WR90 and WR2005 could be associated with an increase in the level of redundancy for the yield estimation, defined in terms of q-information deviation. However, the hydrological change between WR2005 and WR2012 had revealed a reduction in the level of redundant information. This could also be enhanced from Ilunga (2019).

From Table 2, Table 3 and Table 4, generally q-information deviation values were relatively small to be greater than 10%, except for two cases of QCs at the most for each data set. These cases were negligible compared to the rest of QCs in the Middle Vaal basin. In particular, C25D appeared to be the only catchment in the zone of water resource vulnerability for both WR2005 and WR2012. It could mean that no new major hydrological changes occurred in the water resources as far as the different data sets of the Middle Vaal basin were concerned. Similar results were obtained in the case of the Upper Vaal Catchment [3] and could also support the findings of a recent study on the Middle Vaal [5]. This could explain a good approach for the integrated water resource management in South Africa. 

### 4.2. Spatial Distribution of Q-Information/Complexity Deviation

For each data set, as shown in Figure 5, Figure 6 and Figure 7, the pattern of spatial distribution of q-information deviation associated with MARs was not uniformly distributed over the QCs of the different TCs in the Middle Vaal basin. Nonetheless, the spatial distribution of positive and negative q-information deviations was generally approximately in equal proportion. Specific similarity in terms of q-information deviation could be noticed between these figures. It could be believed that both positive and negative q-information deviation values were associated with approximately the same level of MAR dispersion in the different TCs and constituted the dominant metastable status of catchments. This could mean that the proportions of gain and loss in information to reach the Shannon uncertainty could be more or less the same. The proportions of chaotic and null q-information deviation zones were very insignificant compared to the metastable zones in the abovementioned figures.

In particular, in Figure 5 related to data set WR90, the positive and negative iso q-information deviation areas were equally distributed in the North part of the basin. The East, West and Southern cape of the basin were dominated by the positive iso-q information deviation, whereas the negative iso q-information deviation dominated from North towards the Central and West Southern parts of the basin. It is recalled that both iso q-negative and iso q-positive deviation zones were believed resilient in terms of their water resources since q-information deviation were less than the chosen threshold.

For WR90 data set, the QCs with null q-information deviation were found in the East and Southern part of Middle Vaal; however, there were only three QCs. The only chaotic zone was found in the East part of the basin, specifically in C43D. From the results obtained from WR90, the East, West and Southern cape of the basin were characterised by an increase in uncertainty, whereas the North towards the Central and West Southern parts of the basin were characterised by a decrease in information complexity. The former parts could be believed to have lost information while the latter could be associated with gain of information.

In Figure 6, related to data set WR2005, the positive and negative iso q-information deviation areas were equally distributed in the North part of the basin, hence, a similar distribution was noticed for WR90. The East and Southern parts of the basin were dominated by the positive iso q-information deviation, whereas the negative iso q-information deviation dominated the Upper East and West Southern parts of the basin. For WR2005 data set, the QCs with null q-information deviation were found in the Western part of Middle Vaal and Central Eastwards. These QCs were in a double number as compared to those in WR90. The only chaotic zones characterised with high uncertainty were found in the West part of the basin, specifically in C43B and C25D.

As for WR90, the North part could have a balanced situation between decrease and increase in complexity, corresponding to information gain and information loss for WR2005. This is to say that for WR2005, the East and Southern parts of the basin were characterised by an increase in uncertainty, whereas the Upper East and West Southern parts of the basin displayed a decrease in information complexity.

In Figure 7, related to data set WR2012, the proportion of positive iso q-information deviation areas outweighed the negative iso q-information deviation areas in the North part of the basin, unlike the equal distribution of these two categories in WR90. The East, West and Southern parts of the basin were dominated by the positive iso q-information deviation, whereas the negative iso q-information dominated the Central and West Southern parts of the basin. For WR2012 data set, the QCs with null q-information deviation were in a double number as compared to WR90. Areas of information loss as well as information gain areas were not necessarily the same as compared with WR90 data set.

The East, West and Southern parts of the basin were characterised by an increase in information complexity, unlike the Central and West Southern parts of the basin. The only chaotic zones with higher uncertainty were found in the West part of the basin, specifically, QCs C43D and C25D. Areas of lost information and gain of information were not necessarily similar as compared with WR90 and WR2005 data sets.

Figure 5, Figure 6 and Figure 7 showed that the QCs displaying chaotic behaviour were in TCs C43 and C25. Based on Ilunga (2019), high complexity could mean that more information could be needed to reduce the uncertainty associated with the MAR of TCs in the Middle Vaal basin. Thus, supporting water resources management in this basin with sound decision-making could be a priority. It was suggested that these zones could be in hazardous situation when faced with droughts or floods [5].

### 4.3. Hydrological Implications of Iso-Q Information Deviation

For specific QCs in tertiary catchments of the basin, iso q-information deviation areas could be translated into zones that have the same degree of relative stability when compared with the state of equilibrium of hydrological systems. Hence, relatively high positive/negative q-information deviation signals that the catchment as a real system has moved away from its state of equilibrium, which defines the virgin conditions of catchments.

Positive q-information and negative q-information deviation areas were synonymous of being associated with high complexity and low complexity, respectively. According to Ilunga (2019), high complexity could mean that more information could be needed to reduce the uncertainty associated with the MAR of TCs in the Middle Vaal and vice-versa. Similarly, high complexity is associated with low redundancy associated with MARs. Consequently these two categories of q-information deviation yielded indirectly to information loss and information gain for a catchment in the same data set. Strengthening the management and operation of the water resources in the Middle Vaal catchment would be needed, especially in areas of high complexity, i.e., high q-information deviation magnitudes.

Since extreme hydrological events such as floods or droughts are characterised by a considerable amount of uncertainty [3], the high magnitude of q-information deviation (e.g., over the threshold) may signal the tendency towards such events. These events may impact negatively among others the water systems, human life, ecosystem, hydraulic structures, etc. During the occurrence of extreme events, catchments could likely to move from stable conditions (metastability) to unstable conditions (meta-instability). The entropy of the MAR as a contribution from QCs in a given TC associated with higher q-information deviation could be a warning to water resource managers of the state of loss of resilience/sustainability of the water systems in the catchment. Hence, they could explore iso q-information deviation maps in assessing the state of sustainability or vulnerability of the water resources in the different TCs.

Within the same data set, for QCs in a tertiary catchment, iso-q-information deviation depicts the same tendency of relative uncertainty associated with the MAR, therefore, a similar tendency of information complexity or redundancy. Because of the interest in relatively low information redundancy contained in the MAR for the different QCs [5], iso q-information deviation maps could be used in water resources management to determine spatially the areas of unnecessary information, i.e., areas of low complexity. By doing so, reducing the level of redundant information could be cost saving in activities evolved around the water systems. Theoretically, threshold values for iso-q-information deviation are still to be defined with respect to the MAR as the only variable used in this study.

In general, the findings from this study show that the proportion of QCs associated with redundant MAR information was relatively very small as far as the spatial distribution in the form of iso q-information deviation maps was concerned in the Middle Vaal basin. This aspect could be necessary for efficiently managing water resources in this basin. The merit of the study resides in applying entropic measures (i.e., in particular one of the generalised forms of entropy) for a real world data set of high practical interest and impact, specifically in the field of water resource management, which is essential in South Africa, in Africa and the rest of the world. Hence, this study subscribes to the aforementioned field and could be helpful for decision makers to focus on water resource sustainability/resilience or vulnerability at catchment level. Moreover, applications are rarely documented, where catchments are considered, as having nonextensive properties, as far as information related to hydrological variables is concerned.

However, entropy theory does not show exactly the magnitude of the MAR that constitutes redundant information spatially and how it should be explored without impacting aspects of water management.

The current study was preliminarily conducted to explore the Tsallis entropy associated with the MAR as the only variable; the findings could change when variables other than the MAR are introduced. The catchment scale of observation, i.e., quaternary could play a role in the findings of these results.

## 5. Conclusions and Suggestions

The Middle Vaal catchment is one of the prominent areas in Gauteng, which is the main contributing province to the Gross Development Product (GDP) of South Africa. The q-information deviation associated with the MAR, as derived from the Tsallis entropy, was assessed to determine the dynamical level of entropy in the Middle Vaal basin, within the different data sets, i.e., WR90, WR2005 and WR2012. The q-uncertainty parameter varied from 0.5 to about 5, in general, and it did not show any sensitivity beyond this value. It was revealed that MARs for QCs in the TCs of the Middle Vaal basin were generally associated with relatively low q-information deviation magnitude, which implied an acceptable level of water resources resilience in the basin.

The contribution of this study is thought to have introduced the q-information deviation approach of QCs within TCs between 1990 and 2012. An assessment of this approach showed that the proportion of QCs with relatively high complexity (positive q-information deviation with respect to the Shannon entropy) was dominant in the East and Southern parts of the basin for all data sets WR2005, WR90 and WR2012; however, the West part featured in both WR90 and WR2012. Q-information deviation assessment revealed also that the proportion of QCs with relatively low complexity (negative q-information deviation) was dominant in the North towards the Central part and West Southern part of the basin for WR90; while for WR2012, besides the West Southern part of the basin, the Central part was prominent negatively. For WR2005, both the Southern and the Upper East parts displayed dominant negative q-information deformation. 

QCs with null q-information deviation areas were also noticed in all three data sets; however, WR2005 outperformed the remaining two data sets, which scored approximately equally with the same number of QCs with null deviation.

The relatively higher level of entropy associated with the MAR was related to potential chaotic zones in the different TCs. An assessment of the q-information information deviation revealed that the proportion of such zones with relatively high complexity was dominant in the Western part of the basin for WR2012 and WR2005, specifically, in the tertiary catchments C43 andC25, with one chaotic QC each; whereas for WR90, the vulnerability zone was confined to C24, with only one QC. Although WR2012 and WR2005 were affected chaotically in the same TCs, they did not display necessarily the same QCs. The only common vulnerable QC in WR2005 and WR2012 was C25D, while C43D featured in both WR90 and WR2012.

Hence, water managers should pay attention to the situation of relatively higher q-information deviation, especially when perturbations could be associated with hydrological extreme phenomena like floods and droughts. The distributions of MAR defined from the q-information deviation in the form of maps could help decision makers of water systems to understand the dynamical uncertainty change of the MAR within the same data set for an assessment of the runoff as the main contributor to the catchment yield.

It was noticed that the pattern of spatial distribution of q-information deviation associated with MARs was not uniformly distributed over the TCs in the Middle Vaal basin for each data set. Generally, both positive and negative q-information deviation values were associated with approximately the same level of dispersion of the MAR in the different TCs. Generally, q-information deviation values were relatively small and were below the threshold, except in the case of two QCs.

The current study explored interestingly one of the generalised forms of entropy applied to real hydrological data sets of great interest to South Africa and beyond, specifically in water resource management field.

Although q-information deviation was shown to be within the acceptable limits for water resources management in the Middle Vaal between 1920 and 2012, the Tsallis entropy concept does not give any indication on the yield magnitude as far as the MAR is concerned for water resources management, operation and planning.

The limitation of this preliminary study is that it derives its findings from the MAR as the only hydrological variable, and the study has been tested on one basin of the South African water resources. Limiting variables for a given study will always depict a certain degree of information incompleteness. The inclusion of variables other than the MAR could be interesting for new insights in the concept of the q-information deviation defined within the Tsallis entropy context. The extension of this study to basins other than the Middle Vaal could be considered.

In due course, data beyond WR2012 should be taken into consideration as well. The other limitation is to have considered arbitrarily a threshold value for the state of stability criterion of a QC. In principle, a sound methodology should be developed in future for the selection of such a criterion. The change in scale of observation from quaternary to tertiary, or even to secondary, could have an impact on the findings.

## Figures and Tables

**Table 1:** Extracted and adapted from Ilunga (2019), where QCs are summarised in the tertiary catchments for which catchment area, MAR, MAE and MAP are given in the Middle Vaal basin. QC: quaternary catchment; MAR: mean annual runoff, MAE: mean annual evaporation and MAP: mean annual precipitation.

Tertiary Catchment	Quaternary Catchments	Catchment Area (km^2^)	MAE (mm)	MAP (mm)	MAR (WR90) × 10^6^ m^3^	MAR (WR2005) × 10^6^ m^3^	MAR (WR2012) × 10^6^ m^3^	Number of QCs
C24	C24ABCDEFGJH	7512	1291	418	174	154	153	9
C25	C25ABCDEF	7055	1475	418	36	27	37	6
C41	C41ABCDEFGJH	6994	1431	514	317	199	193	9
C42	C42ABCDEFGJHKL	7555	1418	618	226	197	181	11
C43	C43ABCD	2765	1119	306	11	10	22	4
C60	C60ABCDEFGJH	6765	1352	503	166	178	178	9
C70	C70ABCDEFGJHK	6157	1496	555	192	147	155	10

**Table 2:** Q-information deviation characteristics associated with MAR for the QCs for WR90 in the Middle Vaal basin.

Tertiary Catchment	Number of Convergence Point	QCs with D = 0	QCs with D > 0	QCs with D < 0	QCs with Chaotic Zone (D ≥ 0.1)	Number of QCs with Identical Information
C24	1 (A,F), 1 (D,J)	0	3 (AFG)	5 (CJEDBH)	0	3
C25	1 (A,B,C,D,E,F)	0	6 (ABCDEF)	0	0	3
C41	1 (A,B,C,D), 1 (H,J,E),	1H	4 (ACBD)	4 (EFJG)	0	4
C42	1 (K,D,G), 1 (L,H), 1 (C,A), 1 (J,F,B)	1E	5 (ABCJF)	5 (DKLGH)	0	2
C43	1 (A,B,C,D)	0	2 (DB)	2 (AC)	D	0
C60	1 (A,B,C), 1 (GFED)	0	3 (ACB)	6 (EDFGHJ)	0	2
C70	1 (A,B,C,E,G), 1 (DFG)	1D	5 (ABCEG)	4 (FHJK)	0	2
	14	3	28	27	1	14

**Table 3:** Q-information deviation characteristics associated with MAR for the QCs for WR2005 in the Middle Vaal basin.

Tertiary Catchment	Number of Convergence Point	QCs with D = 0	QCs with D > 0	QCs with D < 0	QCs with Chaotic Zone (D ≥ 0.1)	Number of QCs with Identical Information
C24	1 (AFGB), 1 (EH), 1 (J,D)	1 (B)	3 (AFG)	4 (CDJEH)	0	3
C25	1 (ABCDEF)	2 (A,F)	3 (DCE)	1B	1D (0.36; q = 6)	5
C41	1 (ABCD), 1 (FJE)	0	4 (ABCD)	5 (EFGJH)	0	3
C42	1 (KDG), 1 (LH), 1 (CA), 1 (JFB)	1E	5 (ABCJF)	5 (LHKDG)	0	5
C43	1 (ABCD) *	0	1 (B)	3 (DCA)	B	2
C60	1 (GFED), 1 (ABC)	2 (GF)	3 (ABC)	4 (DEHJ)	0	2
C70	1 (DFJ), 1 (ABCEG)	0	5 (ABCEG)	5 (DFJHK)	0	GE
	15	6	24	28	2	21

* 1 (ABCD): 1 stand for one point of convergence between C43A, C43B, C43C and C43D. The rest in the tables is read in a similar way.

**Table 4:** Q-information deviation characteristics associated with MAR for the QCs for WR2012 in the Middle Vaal basin.

Tertiary Catchment	Number of Convergence Point	QCs with D = 0	QCs with D > 0	QCs with D < 0	QCs with Chaotic Zone (D ≥ 0.1)	Number of QCs with Identical Information
C24	1 (AFG), 1 (BDJH)	0	4 (AFGE)	4 (CBDJH)	0	0
C25	1 (ABDEF)	0	5 (ABDEF)	1C	D	2
C41	1 (ABDEF)	0	4 (ABCD)	5 (EFGJH)	0	3
C42	1 (ACFJ), 1 (DKG)	1 (B)	4 (ACFJ)	6 (DEGHKL)	0	4
C43	1 (ABCD)	0	2 (BD)	2 (AC)	D	0
C60	1 (GFED), 1 (ABC)	0	3 (ABC)	6 (DEFGHJ)	0	4
C70	1 (ABCE), 1 (DF)	0	5 (DFJKH)	5 (ABCEG)	0	0
	11	1	27	30	2	13
